# Osteoarthritis and all-cause mortality in worldwide populations: grading the evidence from a meta-analysis

**DOI:** 10.1038/srep24393

**Published:** 2016-04-18

**Authors:** Dan Xing, Yuankun Xu, Qiang Liu, Yan Ke, Bin Wang, Zhichang Li, Jianhao Lin

**Affiliations:** 1 Arthritis Clinic & Research Center, Peking University People’s Hospital, Peking University, Beijing, China

## Abstract

The objective of this study is to investigate the association between osteoarthritis (OA) and all-cause mortality in worldwide populations and to develop recommendations according to GRADE evidence levels. Literature search through Nov 2015 was performed using the electronic databases (including MEDLINE, EMBASE, EBSCO and Cochrane library). The prospective cohort trials that investigated the association between the symptomatic OA (SxOA) or radiological OA (ROA) and all-cause mortality were identified. Hazard ratios (HR) of all-cause mortality in patients with RxOA or ROA were pooled respectively. The evidence quality was evaluated using the GRADE system, while the recommendations were taken according to the quality. Nine of the published literature met the eligible criteria. Meta-analysis revealed that there was no significant difference in the association between SxOA and all-cause mortality (HR = 0.91, 95% CI: 0.68–1.23) and between ROA and all-cause mortality (HR = 1.13, 95% CI: 0.95–1.35). The overall GARDE evidence quality was very low, which will lower our confidence in taking recommendations. To summarize, there was no reliable and confident evidence existed currently in respect of the association between OA and all-cause mortality. Due to the very low level of evidence quality currently, high-quality studies are still required.

Osteoarthritis (OA) is a common disease with rising prevalence, which results in cause of disability among the older people^1^,^2^,^3^. In epidemiology, half of the world’s population aged 65 years or older has OA, which is the most prevalent disorder of articulating joints in humans. It is commonly believed that OA rarely associated with mortality in the elderly and is often considered a physiological condition of ageing^4^. Thus, all-cause mortality had not been a major area of investigation in OA. However, more attention was paid to the association between OA and risk of all-cause mortality recently^5^,^6^.

Although several studies were conducted to validate whether OA is a risk factor for all-cause mortality, there is still in controversy. Nuesch *et al*.^7^ reported that all-cause mortality was found among subjects surveyed from the general population with symptomatic hip or knee OA (SxOA). However, Liu and colleagues examined all-cause mortality in persons with SxOA using data from the Genetics ARthrosis and Progression (GARP) study and Osteoarthritis Care Clinic (OCC) study. The result showed that patients with OA are not at higher risk of death than the general population^8^. Our previous study demonstrated that knee SxOA was associated with an significantly increased risk of all-cause mortality among the people in the rural areas of China and no such association was observed for radiological knee OA (ROA)^6^.

As mentioned above, whether or not OA is associated with an increased risk of all-cause mortality is still conflicting. Therefore, the objective of the present study is to assess the evidence from relevant studies that examine the relation between OA and risk of all-cause mortality in worldwide populations by meta-analysis approach and to develop recommendations according to GRADE (Grading of Recommendations, Assessment, Development, and Evaluation) system^9^,^10^.

## Material and Methods

### Literature search strategy

To assemble all of the potential published studies, Electronic databases including MEDLINE, EMBASE, EBSCO and Cochrane library were searched for all studies published between JAN 1980 and Nov 2015 that investigate the relationship between OA and all-cause mortality in populations. The following MeSH items or free words were taken to balance the search sensitivity and specificity: osteoarthritis, risk, mortality, all-cause mortality, and death rates. Gray studies were identified from the reference of included literature through the manual review. Articles published in languages other than English were also excluded.

### Inclusive and exclusive criteria

Primary studies were considered eligible for inclusion if they met the following criteria:

1. Study design was a prospective cohort study;
2. Studies were included if they investigated the association between the RxOA or ROA and risk of all-cause mortality. SxOA was defined if both pain and ROA were present at the joints;
3. The outcome was all-cause mortality;
4. The hazard ratio (HR) or risk ratio (RR) and its corresponding 95% confidence interval (95% CI) were reported.

No available data, duplicate studies, reviews, rheumatoid arthritis concerned and non-comparable studies were excluded.

### Study selection

Two reviewers (D.X. and Q.L.) independently screened the titles and abstracts for the eligibility criteria. Subsequently, the full text of the studies that potentially met the inclusion criteria were read and the literature was reviewed to determine the final inclusion. Any disagreement was resolved by reaching a consensus through discussion.

### Date extraction

Two researchers (D.X. and K.Y.X.) independently extracted the data from each included literature by the use of a standard data extraction form. The following items were extracted: title, authors, study design, follow-up duration, sample size, age, country, anatomy location, covariates adjusted and all-cause mortality parameters.

### Outcomes

The pooled hazard ratios of all-cause mortality in patients with RxOA or ROA were the outcomes of the present study.

### Assessment of methodological quality

The quality assessment of the included studies was independently conducted by two authors (D.X. and Y.K.X). Disagreements were resolved by discussion or consensus. The methodological quality was assessed using the Newcastle-Ottawa form^11^, which was a valid instrument designed to assess the quality of cohort studies. The Newcastle-Ottawa form assigns a maximum of four points for selection, two points for comparability and three points for exposure or outcome. Newcastle-Ottawa form scores of 7 were considered as high-quality studies and of 5–6 as moderate quality^11^.

### Data analysis

Pooling data was analyzed by STATA 12.0 (Statacorp, college station, Tex). The assessment for statistics heterogeneity was estimated depending on chi-square and I-square test. When the chi-square was p > 0.05, or I-square < 20% indicating low statistical heterogeneity, a fixed effect model was applied. A random effect model was taken when chi-square was p < 0.05, and I-square   > 20%. Furthermore, a sensitivity analysis was carried out by removing one study in each turn, the rest of the studies were analyzed to evaluate whether the results were affected statistically significantly. HR and 95% CI were used to measure the association between OA and all-cause mortality for all studies. If included studies reported several multivariable-adjusted HR, we used the most fully adjusted for potential confounders in the present meta-analysis. RR was used as substitute of HR, when some studies did not directly report HR^12^. A P-value <   0.05 was considered statistically significant. Publication bias was conducted statistically via Begg funnel plots, which measures the degree of funnel plot asymmetry^13^,^14^.

### GRADE Evidence synthesis

The levels of evidence quality were estimated according to the guidelines of the GRADE working group^9^. Although the GRADE system acknowledges the primacy of randomized controlled trials, it also recognizes circumstances in which observational studies generate high quality evidence of treatment effects^15^. A sequential assessment of the evidence quality was applied in GRADE system, and a subsequent judgment on the strength of the recommendations is followed by previous evidence quality assessment. The evidence grades are classified into the four categories: (1) high grade: further research is unlikely to change confidence in the effect estimate; (2) moderate grade: further research is likely to alter confidence in the effect estimate and may change the estimate; (3) low grade: further research is likely to significantly alter confidence in the effect estimate and to change the estimate; and (4) very low grade: any effect estimate is uncertain. The lowest evidence quality for any of the outcomes was used to rate the overall evidence quality according to the GRADE working group. The strengths of the recommendations were taken according to the overall evidence quality. GRADEpro Version 3.6 software was used in grading evidence and taking recommendations.

## Results

### Literature search

**Figure 1:**
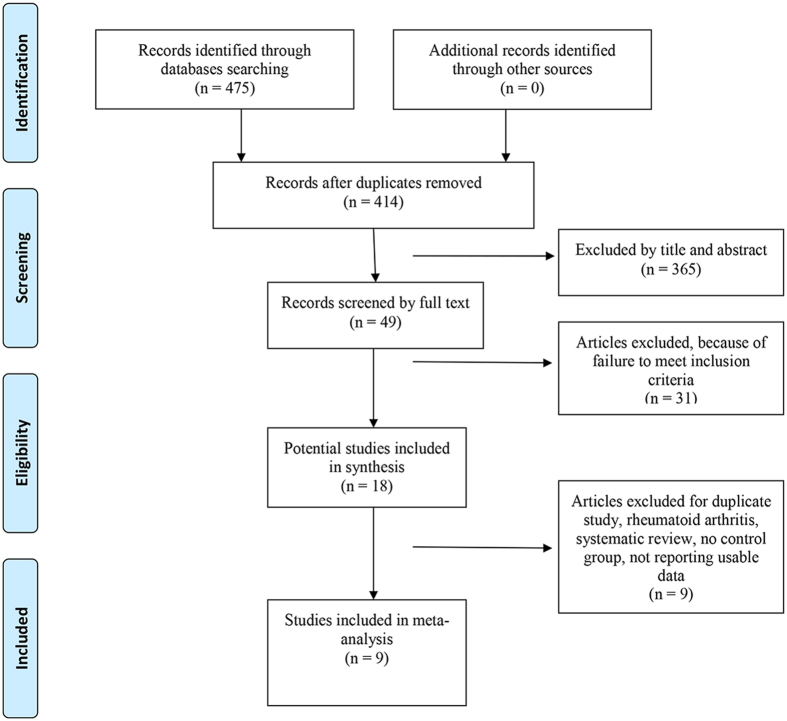
Flowchart of the study selection process.

A total of 414 titles and abstracts were preliminarily identified with the first search strategy, of which 9 of the published literature^6^,^7^,^8^,^16^,^17^,^18^,^19^,^20^,^21^ ultimately met the eligibility criteria (Fig. 1). One of the included literatures contained two trials based on two patients’ databases respectively. All of the included studies investigated the association between the SxOA/ROA and all-cause mortality.

### Study characteristics

The characteristics of the included studies are summarized in Table 1. The individual sample sizes ranged from 181 to 1670 patients with OA. The mean follow-up ranged from 3.9 to 21 years. Six studies were performed to investigate the relationship between the SxOA and all-cause mortality, while other five studies between the ROA and all-cause mortality. All of the included studies had defined the inclusion and exclusion criteria. Only two included studies included both SxOA and ROA patients. There were 3 studies performed in the USA and 1 each in China, Netherlands, England, Italy, Japan and Finland. Two studies recruited patients with knee OA, 2 with hand OA, 1 each with hip OA and hip/knee OA and others with hand, knee, hip, or spine OA. Only one study recruited female patients, while others included both male and female. Only one study conducted two trials in male and female patients respectively.

### Quality assessment and publication bias

Using the Newcastle-Ottawa form, all but three studies were identified as high quality. The results of quality assessment were presented in Table 2. The publication bias was estimated for overall populations by HR of all-cause mortality. No significant publication bias was demonstrated for overall populations by Begg rank correlation method (P = 0.65).

### Meta-analysis

**Figure 2:**
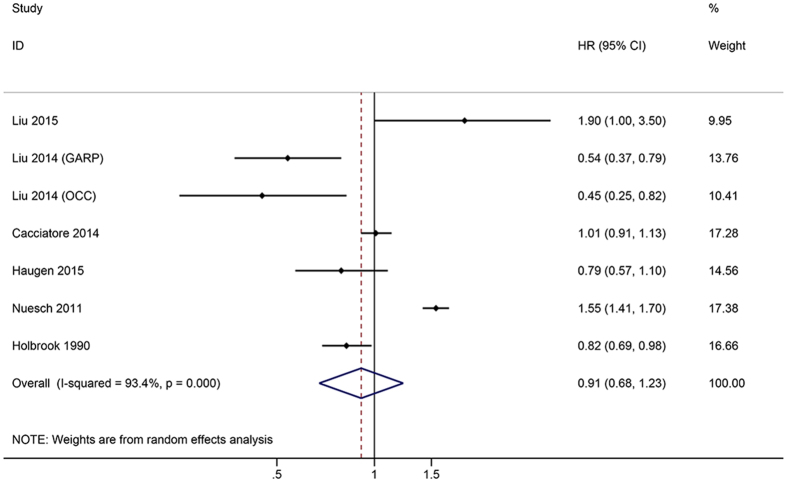
Forest plots showing an association between symptomatic osteoarthritis (SxOA) and the risk of all-cause mortality.

**Figure 3:**
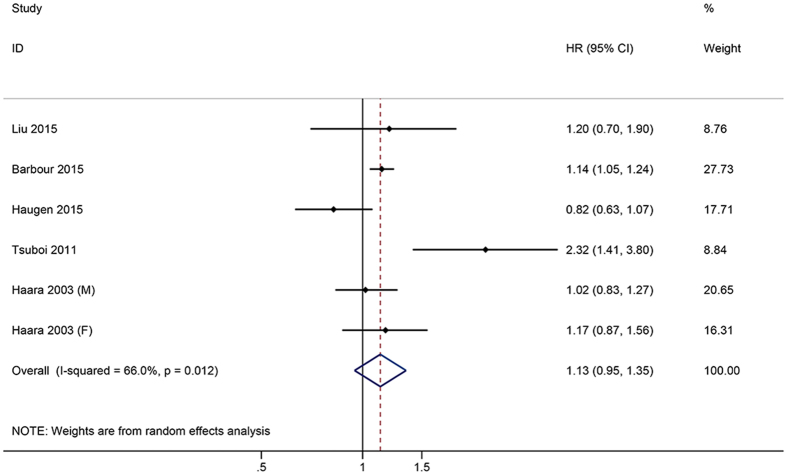
Forest plots showing an association between radiological (ROA) and the risk of all-cause mortality.

In seven studies providing all-cause mortality in RxOA patients, the pooled HR was 0.91 (95% CI: 0.68–1.23). There was no significant difference in HR of all-cause mortality between the SxOA and control groups (Fig. 2). Six trials reported the all-cause mortality in ROA patients. There was no statistically significant difference between the ROA patients and control groups (HR = 1.13, 95% CI: 0.95–1.35) (Fig. 3).

### GRADE Evidence synthesis and recommendation strengths

The above two important outcomes in the present meta-analysis were evaluated using the GRADE system. The level of evidence quality for each outcome was very low (Table 3). Therefore, the level of overall evidence quality was very low eventually. This finding may lower the confidence in any recommendations.

## Discussion

All-cause mortality is a major area of research in OA. However, there has been no consensus regarding whether OA is an independent risk factor for mortality. Furthermore, there have been no recommendations for all-cause mortality in OA patients based on the current evidence. There is a need for an evidence base to help patients or clinician understand the relationship between OA and all-cause mortality and make their clinical decisions. To the best of our knowledge, the present study is the first meta-analysis that uses the GRADE system to evaluate the quality of the evidence investigating the association between OA and risk of all-cause mortality.

To date, nine articles including 10 prospective studies have examined the association between OA and risk of all-cause mortality. Five of them only reported the association between SxOA and mortality, while three between ROA and mortality. Two studies reported the relationship between SxOA/ROA and mortality. In the present study, we conducted meta-analysis according to SxOA and ROA patients respectively. Random effects model was used to conduct statistical analysis because of significant heterogeneity among studies. Thus, more weight was placed on the studies with more sample size. Random effect analysis can lower the risk of bias and get the pooled data conservatively. Although the results showed that SxOA and ROA were not significantly associated with increased risk of mortality, the overall evidence quality was very low. The GRADE evidence quality of pooled results will lower our confidence in clinical decision. The studies were carried out in various durations of follow up, different geographical locations, and with different OA inclusion criteria. The pooled estimate of primary outcomes was consistent across sensitivity analyses and without significant publication bias.

Four included studies^6^,^7^,^16^,^19^ reported the association between SxOA or ROA in the lower limbs and mortality. Because of limited number of included studies involved lower limbs, we cannot conduct subgroup meta-analysis. Although pooled results were not performed in lower limbs, the individual study reported the significant association between the OA in lower limbs and increased risk of mortality, especially in knee SxOA. Lower limbs OA will lower the levels of physical activity, which will influence the survival rate. Therefore, participating in recommended levels of physical activity is associated with reduced risk of mortality^22^. Because of lower evidence quality, we did not have confidence in recommendation currently.

Danielsson *et al*.^23^ reported that death rates were higher than expected in persons with ROA of the knees but not of the hips. However, Cerhan *et al*.^24^ showed that the presence of OA of the hands and bilateral knees was associated with reduced survival, while the presence of OA of the hips and feet was not. The reason might be that reduced survival is attributed to either lower levels of physical activity or the use of medications in patients with OA^24^. In Haara *et al*. study^20^, women with SxOA involving distal interphalangeal joints had an increased mortality, but neither men nor women with ROA of thumb carpometacarpal joint had an increased risk of mortality. Some studies reported that lower levels of physical activity due to disability among the patients with lower limb OA, and the use of non-steroidal anti-inflamatory drugs (NSAIDs) explained the increased risk of mortality from heart disease^6^,^25^. Kerr *et al*.^26^ found that was using NSAIDs associated with an increased risk of mortality from gastrointestinal. In a longitudinal study^27^, the adjusted hazard ratio of celecoxib, diclofenac, meloxicam, rofecoxib and non-selective NSAIDs were 1.39, 1.44, 1.49, 1.58 and 1.76 respectively. Thus, Kerr *et al*.^27^ reported that there was a significant increased mortality risk for those exposed to either COX-2-selective or non-selective NSAIDs.

Our previous study^6^ reported that knee SxOA was associated with an increased risk of all-cause mortality among the residents in the rural areas of China. The reason may be that SxOA patients are likely to suffer from physical disability. Lack of walking disability is one of the risk factors for death from cardiovascular diseases. Furthermore, SxOA patients are commonly take NSAIDs to relieve their joint symptoms, which will increase the risk of morality.

Some degree of clinical heterogeneity was induced by the different anatomical location, medical co-morbidities, ethnicity, genetic background, nutritional status of patients, follow-up duration, and diagnosis criteria for OA. Heterogeneity, to some extent, may have also been caused by study design. One of the major heterogeneity was the several anatomical location of OA during patient recruitment. We cannot perform subgroup analysis according to anatomical location due to two studies in knee, one in hip and others in knee, hip, hand or spine. Because of limited information got from original studies, heterogeneity cannot be completely resolved.

Although the above kinds of heterogeneity may palace an influence on the pooled results of meta-analysis, the evidence quality was summarized according to the current evidence. Because of clinical heterogeneity and limited information, we, therefore, drew the conclusion that the GRADE evidence quality was very low. The present evidence quality meant that reliable and confident evidence did not exist currently in respect of the association between OA and all-cause mortality. Moreover, the very low GRADE evidence quality need further high-quality studies to be complemented.

The methodological quality assessment for original studies identified a number of limitations to the current evidence base. (1) The diagnosis of osteoarthritis in some studies^20^,^21^ was recorded by a general practitioner and was not validated based on published classification criteria. (2) Some individuals with asymptomatic osteoarthritis would be misclassified into the control group, which will lead to a bias^21^. (3) The various duration of follow-up would place a bias on the current results. (4) Clinical heterogeneity may be caused by the preexisting conditions of the patients, various diagnosis for OA, the experience level of the evaluators, severity of OA, medical commodities, smoking and the economic level. The above confounding factors might have an impact on the present outcomes. (5) Meta-analyses of observational studies are subject to bias and provide inappropriate estimates for effects size when compared to interventional studies such as RCT. While the results of this meta-analysis should be considered appropriate, these limitations should be considered when interpreting the findings.

In the GRADE system of rating the quality of evidence, each observational study began as low quality evidence but was rated up by three categories of items. Firstly, the included studies did not yield large or very large estimates of the magnitude of an exposure effect, we may not be confident about the results. Secondly, there was no evidence that the influence of all plausible confounding would reduce a demonstrated effect or suggest a spurious effect. Thirdly, an exposure-response gradient was not present, accordingly upgrade the quality of evidence was not for this outcome. We consider that the above factors could significantly influence the stability of the outcomes. The above factors of GRADE evidence quality did not account for the upgraded evidence quality. Therefore, the overall GRADE evidence quality was very low. It indicates that the there is no exact evidence involving the association between OA and all-cause mortality. High-quality clinical trials are required to verify the above area of OA. To some extent, the present study is meaningful for taking clinical recommendation.

According to the very low GRADE evidence quality, the present meta-analysis of cohort studies showed that reliable and confident evidence do not exist currently in respect of the association between OA and all-cause mortality. The increasing prevalence of OA worldwide calls for further high-quality studies to investigate the effects of OA on mortality.

## Additional Information

**How to cite this article**: Xing, D. *et al*. Osteoarthritis and all-cause mortality in worldwide populations: grading the evidence from a meta-analysis *Sci. Rep.* **6**, 24393; doi: 10.1038/srep24393 (2016).

## Figures and Tables

**Table 1:** Characteristics of included studies.

**Study**	**Year**	**Country**	**Year of baseline survey**	**Age**	**Male (M/F)**	**Anatomy location**	**Follow-up (y)**	**Covariates adjusted for**
Liu *et al*.	2015	China	2005–2013	>50	505/520	Knee	8	Age, sex, BMI, income level, education, levels of occupational physical activity and comorbidities
Barbour *et al*.	2015	US	1988–2013	>65	0/9704	Hip	16.1 ± 6.2	K/L grade, Croft grade, age
Liu *et al*. (GARP)	2015	Netherlands	2000–2011	60	69/314	hand, knee, hip, or spine	9.9	–
Liu *et al*. (OCC)	2015	Netherlands	2005–2011	61	55/404	hand, knee, hip, or spine	3.9	–
Cacciatore *et al*.	2014	Italy	1992–2004	73.8 ± 6.6	554/734	hand, knee, hip, or spine	12	Age, sex, BMI, Heart rate, NSAIDs, Frailty and comorbidities
Haugen *et al*.	2015	US	1990–2011	62.2 ± 8.2	623/725	hand	21	Age, sex, cohort, BMI, total cholesterol:HDL ratio, current lipid lowering treatment, increased blood pressure, current antihypertensive treatment, elevated fasting or non-fasting blood glucose, current antidiabetic treatment (oral or insulin), current use of NSAIDs, daily use of aspirin, current/previous smoking, alcohol use
Nuesch *et al*.	2011	England	1994–2009	>35	503/660	Hip or Knee	14	Age, gender
Tsuboi *et al*.	2011	Japan	1997–2007	>60	329/460	knee	10	Age, gender, BMI, and lifestyle
Haara *et al*.	2003	Finland	1978–1994	>30	1560/2035	Hand	15–17	Age, education, physical stress at work, BMI, and smoking
Holbrook *et al*.	1990	US	1974–1986	>50	234/285	hand, knee, hip, or spine	12	Age

M: male, F: female, BMI: body mass index, K/L: Kellgren/Lawrence scale, y: years, NSAIDs: non-steroidal antiinflammatory drugs.

**Table 2:** Study Quality assessment using Newcastle-Ottawa scale for cohort studies.

**Study, Year**	**Selection**				**Comparability of cohorts (matched for)**	**Outcome**			**Total score**
	**Representativeness of exposed cohort**	**Selection of nonexposed cohort**	**Ascertainment of exposure**	**Outcome not present at baseline**		**Assessment of outcome**	**Sufficient follow-up duration**	**Adequate follow-up**	
Liu^6^	*	*	*	*	** (age, gender, etc)	*	*	*	9
Barbour^16^	*	*	–	*	* (K/L grade, Croft grade, age)	*	*	*	7
Liu^6^	*	*	–	*	–	*	*	*	6
Cacciatore^18^	*	*	*	*	–	–	*	*	6
Haugen^17^	*	*	*	*	** (age, gender, etc)	*	*	*	9
Nuesch^7^	*	*	*	*	** (age, gender, etc)	*	*	*	9
Tsuboi^19^	*	*	*	*	** (age, education, etc)	*	*	*	9
Haara^20^	*	*	*	*	** (age, education, etc)	*	*	*	9
Holbrook^21^	*	–	*	*	* (age)	–	*	*	6

**Table 3:** The GRADE evidence quality for each outcome.

**Quality assessment**							**Quality**	**Importance**
**No of studies**	**Design**	**Risk of bias**	**Inconsistency**	**Indirectness**	**Imprecision**	**Other considerations**		
All-cause mortality in ROA								
6	observational studies	serious	serious	no serious indirectness	serious	none	⊕○○○ VERY LOW	CRITICAL
All-cause mortality in SxOA								
7	observational studies	serious	serious	no serious indirectness	serious	none	⊕○○○ VERY LOW	CRITICAL
